# The role of iconic gestures and mouth movements in face-to-face communication

**DOI:** 10.3758/s13423-021-02009-5

**Published:** 2021-10-20

**Authors:** Anna Krason, Rebecca Fenton, Rosemary Varley, Gabriella Vigliocco

**Affiliations:** Division of Psychology and Language Science, University College London, 26 Bedford Way, London, WC1H 0AP UK

**Keywords:** Multimodal communication, Iconic gestures, Mouth movements, Word recognition

## Abstract

Human face-to-face communication is multimodal: it comprises speech as well as visual cues, such as articulatory and limb gestures. In the current study, we assess how iconic gestures and mouth movements influence audiovisual word recognition. We presented video clips of an actress uttering single words accompanied, or not, by more or less informative iconic gestures. For each word we also measured the informativeness of the mouth movements from a separate lipreading task. We manipulated whether gestures were congruent or incongruent with the speech, and whether the words were audible or noise vocoded. The task was to decide whether the speech from the video matched a previously seen picture. We found that congruent iconic gestures aided word recognition, especially in the noise-vocoded condition, and the effect was larger (in terms of reaction times) for more informative gestures. Moreover, more informative mouth movements facilitated performance in challenging listening conditions when the speech was accompanied by gestures (either congruent or incongruent) suggesting an enhancement when both cues are present relative to just one. We also observed (a trend) that more informative mouth movements speeded up word recognition across clarity conditions, but only when the gestures were absent. We conclude that listeners use and dynamically weight the informativeness of gestures and mouth movements available during face-to-face communication.

**Supplementary Information:**

The online version contains supplementary material available at 10.3758/s13423-021-02009-5.

## Introduction

Face-to-face communication is a well-orchestrated process of exchanging multimodal information under various, sometimes challenging, conditions (e.g., a chat between friends in a noisy restaurant). Here, we investigate how iconic gestures and facial movements affect spoken word recognition under clear and distorted listening conditions by asking how listeners’ use of these cues depends upon their informativeness.

## Iconic gestures

Iconic gestures that imagistically evoke features and properties of concepts (e.g., clenching one’s fist and moving the arm up and down to express a hammering action) are common in face-to-face communication. For example, 20% of the utterances in dyadic interactions, in which adults spontaneously talk about a set of known and unknown objects (Vigliocco et al., 2021), contain iconic gestures, whereas only 10% of the produced utterances contain beat gestures. Iconic gestures are processed automatically, as clearly demonstrated by the fact that listeners attend gestures even when they are misleading (Green et al., 2009; Habets et al., 2010; Kelly et al., 2014; Kelly et al., 2010; McNeill et al., 1994; Willems et al., 2009; Wu & Coulson, 2007). For instance, McNeill et al. (1994) showed participants video clips of a speaker telling a cartoon story accompanied by either matching or mismatching iconic gestures; finding that participants considered the information from both types of gestures when asked to recall the story. In another study, Kelly et al. (2010) presented participants with action primes followed by either congruent, weakly incongruent, or strongly incongruent speech–gesture video presentations. The participants’ task was to decide whether the speech or gesture from a video was related to an action prime seen earlier. The authors found that individuals made fewer errors for the presentations including weakly incongruent gestures (e.g., saying ‘chop,’ and gesturing ‘cut’), compared to strongly incongruent gestures (e.g., saying ‘chop,’ and gesturing ‘twist’), further suggesting that people make use of all the information available even when the meaning the gestures evoke mismatches the speech. Recent studies have extended these findings by showing that incongruence between speech and a visual cue can be especially detrimental for people with aphasia (Vigliocco et al., 2020) and by demonstrating similar interactions between different channels (hand and mouth) in users of British Sign Language (Perniss et al., 2020).

Integration of auditory and gestural information has been assessed using, for example, gestures containing information not present in speech (Beattie & Shovelton, 1999; Cocks et al., 2009; Cocks et al., 2018; Kelly et al., 1999) or degraded speech to increase difficulty (Holle et al., 2010; Obermeier et al., 2012). For example, Holle et al. (2010) tested comprehension of audiovisual sentences (with or without gestures) with different signal-to-noise ratios (SNR) and asked participants to type down all the information they understood. Participants were able to recall more information when the gestures were present indicating that gestures can aid speech comprehension especially in adverse listening conditions. Obermeier et al. (2012) further found that this gestural enhancement occurs under difficult listening conditions regardless of whether the challenge is due to external noise or hearing impairment.

Gesture presence can support speech comprehension by virtue of enhancing semantic activation (McNeill, 1992, 2000; Morrel-Samuels & Krauss, 1992). If this is the case then, the degree of informativeness of the gesture (i.e., the extent to which one can recognize the gesture) will matter.

## Mouth movements

Facial (especially mouth) movements are among the visual cues that are almost always available in face-to-face communication, and it is well-known that they affect speech perception (McGurk & MacDonald, 1976). Seeing mouth movements makes speech recognition easier (Peelle & Sommers, 2015) by reducing lexical competition (Jesse & Massaro, 2010; Lachs & Pisoni, 2004; Tye-Murray et al., 2007), especially in noisy listening conditions (Drijvers & Özyürek, 2017; Ma et al., 2009; Reisberg et al., 1987; Ross et al., 2007; Schwartz et al., 2004; Sumby & Pollack, 1954). For example, Tye-Murray et al. (2007) employed a repetition task with stimuli distorted by speech babble presented in auditory-only, visual-only, or audiovisual combinations. They found that performance was enhanced for audiovisual presentations. Moreover, people benefit from visible speech in clearly audible conditions, in particular when the complexity of a message increases. For instance, Arnold and Hill (2001) measured participants’ comprehension of connected speech by presenting short stories that varied in their difficulty (e.g., a passage uttered in a non-native accent) and modality (either auditory-only or audiovisual). Participants performed better when mouth movements were present, replicating Reisberg et al. (1987), and suggesting that the information from mouth movements is automatically processed with speech.

In contrast to iconic gestures, mouth movements are primarily useful in decoding the phonological information and listeners benefit from audiovisual speech because facial gestures can support predictions for upcoming words (Solberg Økland et al., 2019). This has been captured by the notion of *visemes*—that is, the shape(s) of the lips that correspond to a particular phoneme or group of phonemes (Fisher, 1968; Massaro & Cohen, 1995). For example, sounds that are produced more anteriorly on the mouth, such as /f/, are visually more distinct than phonemes with a more posterior place of articulation, such as /k/, and hence inform the listener to a larger extent (Massaro et al., 1993). However, visemes provide only limited information about voicing and manner of articulation and lack a one-to-one correspondence with phonemes (/f/ and /v/ are indistinguishable in the visual context). Moreover, visemes can be different when isolated sounds are produced and when they are co-articulated (e.g., /b/ in ‘bean’ and ‘bow’). Here, we develop quantitative measures of mouth informativeness for English words, rather than employ a priori categories, to operationalize the amount of information available in speakers’ mouth movements.

## Weighting the multimodal cues

The majority of previous studies have only looked at the impact of one visual cue: iconic gestures or mouth movements, while the other cue was eliminated to achieve control. Thus, the face is cropped or covered in studies of gestures (e.g., Drijvers & Özyürek, 2017; Habets et al., 2010; Hirata & Kelly, 2010; Holle & Gunter, 2007; Holle et al., 2010), and the hands are not visible in studies of audiovisual speech (e.g., Ross et al., 2007; Solberg Økland et al., 2019; Tye-Murray et al., 2007). Only a handful of studies have investigated both gestures and mouth movements (Drijvers & Özyürek, 2017, 2020; Drijvers et al., 2019; Hirata & Kelly, 2010; Skipper et al., 2009; Zhang, Ding, et al., 2021a; Zhang, Frassinelli, et al., 2021b).

For example, Drijvers and Özyürek (2017) presented participants with video clips of a speaker uttering words and producing gestures. Mouth movements were visible or blurred, and the speech was clear or degraded. Participants had to report the produced words. The researchers found that subjects benefited most from a double enhancement (i.e., when both cues were present), especially when the speech was moderately degraded, replicating previous studies (Ma et al., 2009; Ross et al., 2007). Crucially, they also found that iconic gestures affected word comprehension to a larger extent than mouth movements. However, we do not know how informative the mouth movements were in the study.

In another study, Zhang, Frassinelli, et al. (2021b) looked at brain activity during audiovisual connected speech processing. The researchers measured changes in the N400 amplitude—a negative event-related potential associated with semantic processing (Kutas & Federmeier, 2011)—by looking at word predictability, prosodic stress, iconic gestures, beat gestures, and mouth informativeness. They found that all the multimodal cues modulated the N400 amplitude along with word predictability, although the degree to which they did so depended on the presence of other cues. For mouth informativeness, they found that it enhances speech perception when iconic gestures are also present, similarly to the double enhancement effect found in Drijvers and Özyürek (2017).

## Current study

The goal of the present study was to address how iconic gestures and mouth movements modulate word recognition in a picture–word matching task (i.e., we presented pictures of objects or actions followed by video clips of a speaker saying and gesturing a word). We manipulated the presence of gestures and their congruency with the spoken words, as well as the clarity of the speech (clear or moderately degraded), but we kept mouth movements always visible, as it is in face-to-face contexts. In contrast to previous studies, we used measures of informativeness of both the gesture and the mouth movement obtained in norming experiments as predictors. Employing these measures is a more ecologically valid and novel (for mouth movements) way of assessing the impact of multimodal cues on speech processing and can further inform our understanding of the underlying mechanisms without eliminating (e.g., blurring or covering) one of the visual cues and thereby removing information about their possible interactions.

On the basis of prior research, we predicted the following:

(i) *Congruent gestures versus no gestures.* Performance should be enhanced when iconic gestures are presented alongside speech. This should be the case in particular in the degraded speech conditions, when meaning is harder to decode from auditory information alone. More informative mouth movements should also be useful in the degraded speech condition, especially in the absence of gestures (provided that gestures and mouth movements influence word recognition to a different extent; Drijvers & Özyürek, 2017) or in addition to gestures (provided that the presence of both cues enhance comprehension to a larger degree than the presence of a single cue; Drijvers & Özyürek, 2017).
(ii) *Incongruent gestures versus no gestures.* Performance should be hindered when incongruent iconic gestures are present provided that they are processed automatically alongside speech (Kelly et al., 2010; McNeill et al., 1994). This will be the case particularly for the degraded speech. The effect (if any) of mouth movements will be difficult to document because of the large interference effect from the gestures.
(iii) *Congruent versus incongruent gestures.* Performance should be significantly better for congruent relative to incongruent gestures, particularly when congruent gestures are more informative. Performance will be most disrupted when incongruent, highly informative gestures are present, and speech is degraded. Iconic gestures accompanied by more informative mouth movements should have a greater effect on word recognition than a single cue alone, especially when the speech is degraded (Drijvers & Özyürek, 2017).

## Methods

### Norming studies

#### Gesture informativeness norms

Forty-five native English speakers (28 females; *M* = 27 years, *SD* = 6.2) were recruited using Prolific (http://www.prolific.co/). Participants had normal or corrected-to-normal vision and hearing and did not report any known neurological or psychiatric conditions. All participants consented to participate in the study and received payment on completion according to Prolific policy. The study received ethical approval (Research Ethics Committee [0143/003]) from UCL.

**Fig. 1:**
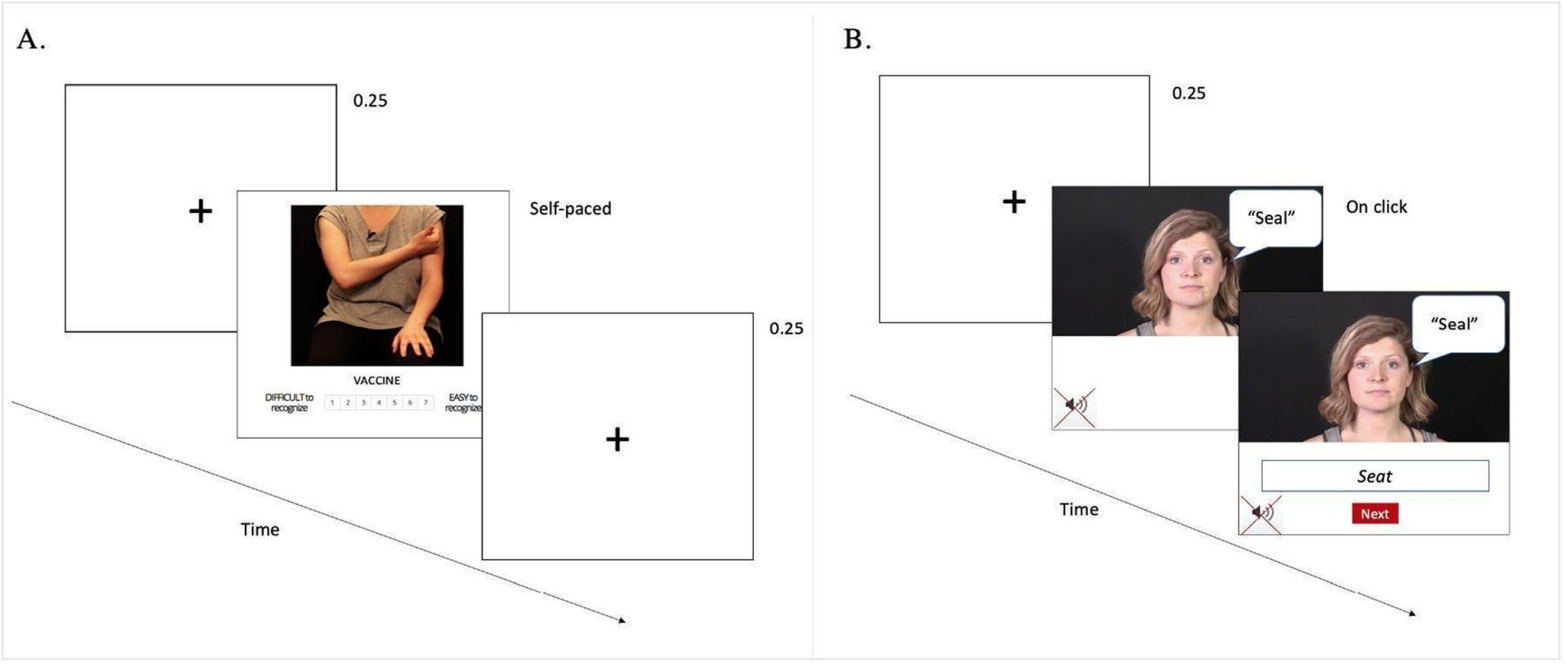
Example trial of the gesture (**a**) and mouth **(b)** informativeness tasks

The materials for this study were collected simultaneously with the materials for the main experiment. We video-recorded a female native-English speaker uttering and gesturing 187 concrete, gesturable words in isolation (mean length of a clip was 2 seconds) in a professional recording studio at UCL. Each word was recorded twice: with and without gestures. For the former, the model was asked to produce gestures as naturally as possible and place her hands on her lap when finished; for the recording without gestures, the model was prompted to keep her hands in her laps. The model wore neutral-colored clothes, wore no makeup, and was sitting on a chair against a unicolored background. For this norming study, only the videos where the gesture was present were used and further edited using iMovie (Version 10.1.12) such that only hand actions remained visible, and audio was muted (see Fig. 1a).

Participants took part in an online experiment designed using Gorilla (https://gorilla.sc/). The task, previously used in other gesture studies (e.g., Drijvers & Özyürek, 2017), was to rate on a scale from 1 to 7 how well the gestures represented the written words displayed on the screen (with 1 being *very difficult*; 7 being *very easy to recognize*). Each participant responded to 93–94 items randomly selected from the whole corpus. There were also two practice trials at the beginning of the experiment. In addition, 20 filler (*M* = 1.49, *SD* = 1.00) items were randomly presented during the experiment to ensure that participants used all the available ratings. The fillers consisted of the gestures that did not match the written words on the screen (e.g., the gesture represented a ‘hammer,’ and the written word was ‘vaccine’) and were not included in the analysis. Participants were allowed to take three breaks during the experiment but were asked to complete the study within 40 minutes. The trials were self-paced, and there was a fixation cross of 250 ms prior to each trial.

The participants’ responses had a grand mean of 5.24 (*SD* = 1.27), suggesting that most of the selected iconic gestures matched well with the written form. The gesture informativeness is the mean rating score, with bigger values (i.e., closer to 7), signifying that the gesture is more informative.

#### Mouth informativeness norms

We recruited 145 monolingual native English speakers using Prolific (http://www.prolific.co/). Eight participants were removed from the analysis: three participants experienced technical difficulties during the experiment, three timed out, and the last two did not respond correctly to the ‘catch trials’ (see paragraph below). The remaining 137 participants (71 females, 64 males, and two nonbinary; *M* = 29 years, *SD* = 6.24) reported normal or corrected-to-normal hearing and vision and had no known neurological or psychological disorders. All participants consented to participate in this online study and were paid for their time according to the Prolific policy. Ethical approval was obtained from the UCL Research Ethics Committee (0143/003).

We recorded 745 muted video clips in which an English-speaking actress produced single words. The selected words were concrete (ratings between 3.93 and 5 on a 5-point scale; Brysbaert et al., 2014) and referred to either everyday objects (e.g., ‘ball’), living beings (e.g., ‘fish’), actions (e.g., ‘watching’), or attributes (e.g., ‘hot’). The videos were recorded in a soundproof recording studio at UCL and contained only the face of the speaker presented on a dark unicolor background (see Fig. 1b). The video stimuli were randomly divided into seven lists, and each participant was randomly assigned to complete one of the lists. Additionally, we selected 12 pictures from various open-source platforms that served as catch trials presented on random occasions. In the catch trials, participants saw briefly presented images followed by a question about the picture (e.g., *Was that a candle?*). This was to ensure participants paid attention throughout the task.

Participants were invited to take part in an online lipreading task created with Gorilla (https://gorilla.sc/). They were instructed to watch silent video clips (mean length of 1 second) presented on a screen and then type their guess of what was uttered by the speaker. The same video was successively played twice to ensure subjects did not miss any trials and were able to extract the available information from the lips. After the second presentation, a blank answer box appeared below the video (see Fig. 1b). The task was self-paced. Before each trial, a fixation cross was presented on the screen for 250 ms. Participants were prompted to type a single word response using lower case letters and to avoid spaces. They were encouraged to make their best guess if they were unsure. Prior to the experimental trials, subjects performed seven practice trials followed by feedback.

We operationalized informativeness using the phonological distance between the typed responses and the target in the following manner. First, we converted written responses to phonetic transcription (International Phonetic Alphabet [IPA]) using available online software (https://tophonetics.com/). We then corrected accidental spaces and arbitrarily assigned the lowest value of informativeness to any missing answers (e.g., blank or ‘I don’t know’) to reflect the level of difficulty these words posed (62 trials out of 14,514). Second, we used the *PanPhon* package (Mortensen et al., 2016) in PyCharm 2018.2.4, which consists of a large database of phonemes and their phonological features, to calculate *feature editdistance*. This is a string edit distance with weighted phonological features^1^ divided by the maximum length of a given word. The calculated distance was normalized and ranged from 0 to 1 (*M* = 0.49, *SD* = 0.16). The measure of mouth informativeness for a given word is, therefore, the mean distance value, with a smaller distance (i.e., closer to zero), corresponding to a larger informativeness score.

### Main study

#### Participants

A total of 104 native English speakers (*M* = 29 years, *SD* = 6.95, 65 females) were recruited via Prolific (http://www.prolific.co/). All were right-handed monolinguals, who reported no language impairments and had normal or corrected-to-normal hearing and vision. As in the norming studies, all participants provided their consent for participation and were paid for their time under UCL ethical approval (0143/003). We were unable to conduct sample size calculations a priori based on effect sizes because of a lack of studies from which relevant information could be derived. Note, however, that according to Trafimow (2018), a study of 104 participants (>50 in each between-subject group) has between ‘good’ to ‘excellent’ probability of replication and ‘moderate’ precision.

#### Materials

**Fig. 2:**
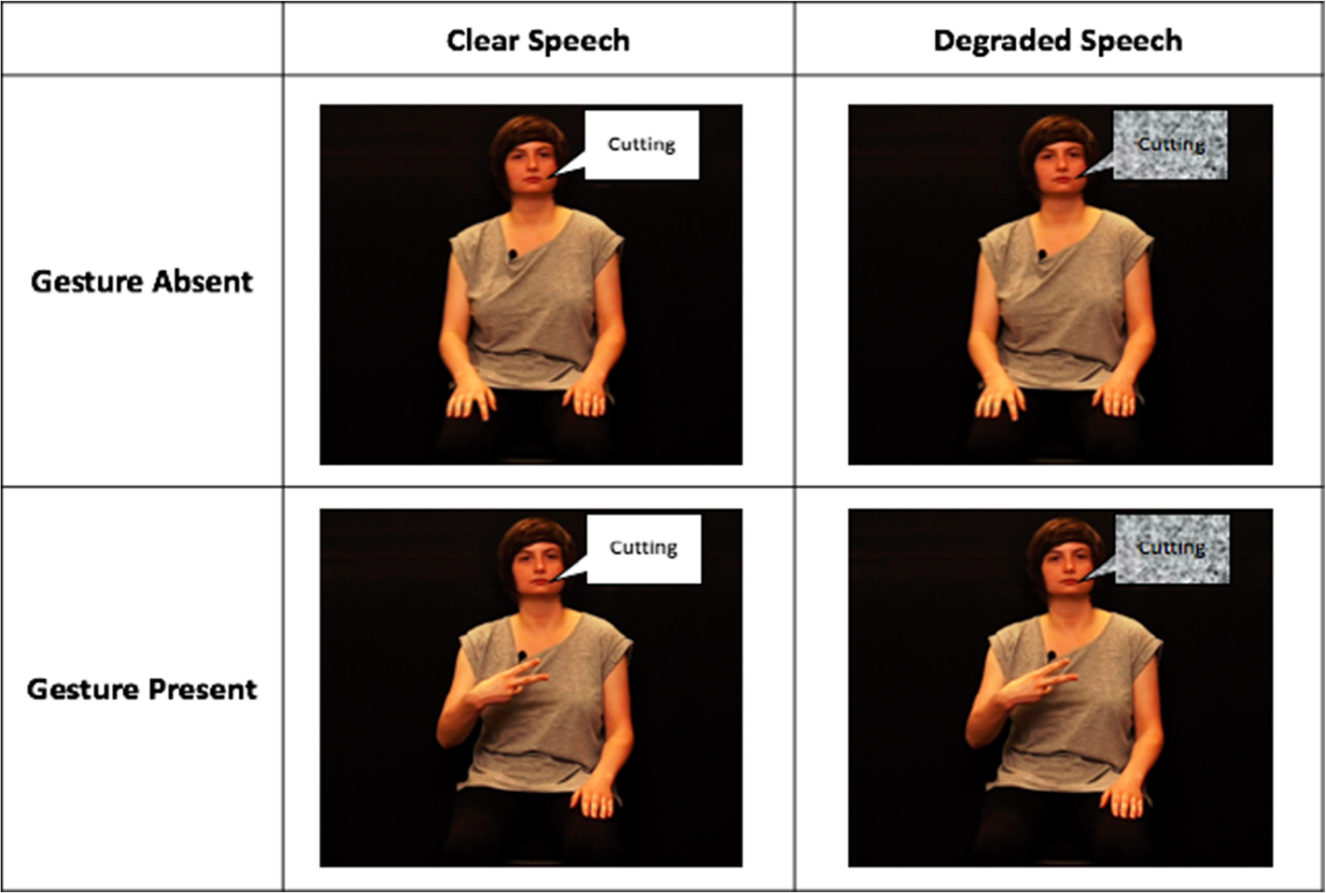
Schematic representation of the manipulations used in the main experiment. In this example, the gestures are congruent with the speech

Materials consisted of 120 gesturable target words referring to either actions (e.g., ‘watching’) or objects (e.g., ‘ball’) that varied in their mouth (*M* = 0.52, *SD* = 0.13, range: 0.17–0.87) and gesture (*M* = 5.30, *SD* = 1.25, range: 1.67–6.92) informativeness based on the results from the norming studies; 120 video clips recorded as a part of the gesture informativeness norming experiment with visible face, body, and hands of the actress (see Fig. 2); and 240 monochromatic pictures: one matching and one mismatching the target word. For the mismatching pairs, we avoided words that shared phoneme onsets as well as words for which the corresponding limb gestures resembled each other. The pictures were taken from various sources, including Druks and Masterson (2000), Snodgrass and Vanderwart (1980), and other online platforms.

We manipulated the clarity of the auditory signal to create conditions more similar to those in everyday interactions, in which comprehenders may rely more on visual cues such as gestures and mouth movements. We included a ‘clear’ (unedited) condition, as well as a six-band pass-filter vocoded condition with maintained rhythmic structure but reduced pitch-related information (Shannon et al., 1995). Six-band filtering was chosen because it has been shown to moderately hinder speech comprehension (Drijvers & Özyürek, 2017). To manipulate the sound files, we used the same technique as described in Drijvers and Özyürek (2017), following a custom Praat script (Boersma & Weenink, 2021).

We also manipulated the presence of gestures to assess whether mouth movements enhance comprehension in addition to the iconic gestures and whether the effect of gestures is larger than mouth movements (Drijvers & Özyürek, 2017). Congruency between gestures and speech was additionally manipulated in separate blocks presented to different participants in order to avoid the possibility that mixing congruent and incongruent gestures would lead to the use of strategies (e.g., such as ignoring the gestures altogether). The condition in which stimuli had congruent gestures or no gestures is more ecologically valid, given that in real-world communication, gestures are not always present, but when they are, they are congruent with the speech. The condition in which stimuli have incongruent gestures or have no gestures provides a less ecologically valid scenario. However, this manipulation establishes to what extent participants automatically process gestures even when they should strategically ignore them because of interference (Kelly et al., 2010).

To create the incongruent speech–gesture pairs, we used the procedure introduced by Perniss et al. (2020) and Vigliocco et al. (2020), in which the head from one video was cropped (together with the auditory signal) and combined with the body from another (muted) video. We additionally edited the congruent video pairs in a similar way, i.e., we cropped the head from a speech-only video and pasted it on the corresponding speech–gesture video with an aligned audio file to ensure consistency across congruent and incongruent stimuli. All video manipulations were done in iMovie (Version 10.1.12). Furthermore, we constrained the selection of the incongruent speech–gesture pairs in the following way: (i) paired items had the same syllable length but differed at least in phoneme onsets (e.g., ‘walking–bowling’), (ii) associated gestures of the paired items did not resemble each other (e.g., excluding pairings such as ‘bowling–throwing’), and finally, (iii) action and object items could not be paired together (e.g., excluding ‘throwing–airplane’).

Overall, participants saw congruent or incongruent speech–gesture videos under four possible manipulations: (i) clear, gesture absent (where speech is clear and not accompanied by gestures), (ii) degraded, gesture absent (where speech is noise-vocoded and not accompanied by gestures), (iii) clear, gesture present (where speech is clear and accompanied by gestures), and finally, (iv) degraded, gesture present (where speech is noise-vocoded and accompanied by gestures; see Fig. 2). In all the conditions, mouth movements were present as in naturalistic face-to-face communication settings. For informativeness scores, picture materials, and Praat script, please see https://osf.io/gudj6/. For audio/video materials, please contact the corresponding author.

#### Procedure

After consenting to take part in an online computer-based experiment developed using Gorilla platform (https://gorilla.sc/), participants were randomly allocated to one of the two experimental groups: congruent (53 participants) or incongruent (51 participants)*.* Each trial started with a fixation cross (250 ms) followed by an interval (300 ms) that preceded the onset of the picture. An image was then presented for 1,000 ms, and a video clip would play automatically on the next screen with the simultaneous presentation of the ‘YES’ and ‘NO’ answer boxes below. Participants’ task was to decide whether the spoken words uttered by the speaker in the videos matched previously seen pictures of an object/action by selecting (as accurately and as quickly as possible) one of the answer boxes using the mouse. Participants could respond during the presentation of the videos to ensure that the reaction times (RT) measured in this study captured the moment of meaning recognition. Participants were presented with the same video stimulus twice: once with a matching target image (*YES trials*) and once with a mismatching image (*NO trials*), completing 240 trials in addition to eight practice trials (not seen elsewhere) prior to the experiment. The main trials were randomly divided into four blocks of 60, between which participants could take a self-paced break. The experimental blocks were also randomized across participants. Additionally, we introduced eight ‘catch trials’ (two per block, randomly presented) to ensure participants paid attention to the videos. The catch trials consisted of pictures (different from those used for the target items) briefly presented on the screen, followed by a picture-verification question (e.g., *Was that a dog?*).

#### Data analysis

Generalized logistic and linear mixed-effects regression analyses, with Holm’s corrected pairwise comparisons where necessary, were performed in RStudio (RStudio Team, 2015) using *lme4* package (Bates et al., 2015). Mixed-effect regression was used to handle categorical and continuous variables without loss in power, as well as non-independence in the data (Dixon, 2008; Jaeger, 2008; Meteyard & Davies, 2020). It is also more suitable for unbalanced designs, can easily accommodate missing data, and can account for both by-subject and by-item variance (Gelman & Hill, 2006; Meteyard & Davies, 2020). We carried out two separate analyses (Analysis 1 and Analysis 2), both assessing participants’ accuracy (binomial dependent variable) and RT (continuous dependent variable). In both sets of analyses, we focused only on the trials where the spoken word and the picture matched (*YES trials*) to ensure reliability (Stadthagen-Gonzalez et al., 2009), following Vigliocco et al. (2020). Prior to the analyses, outliers were identified as (i) any participant with an accuracy below three standard deviations or RT above three standard deviations from the mean; (ii) any item with an accuracy below chance level (50%) or RT above three standard deviations from the mean; (iii) any trial with RTs greater than three standard deviations from the mean of all trials to ensure normal distribution; (iv) any trials which had video loading issues signaled by Gorilla. Outliers (~10% of the data) were further removed from the analyses (see the Supplementary Materials for a full description of the outliers).

In Analysis 1, we ran separate models for congruent and incongruent gestures entering the following fixed effects: gesture presence, speech clarity, and mouth informativeness, as well as all possible interactions between them (up to a three-way interaction) into the model. In Analysis 2, we selected all the trials in which the gesture was present across the congruent and incongruent conditions and included the following fixed effects in a new set of models: speech clarity, mouth informativeness, gesture informativeness, congruency, and up to three-way interactions between them. Taking a design-driven approach (Barr et al., 2013), we entered intercepts for subjects and items as random effects; we also entered by-subject and by-item random slopes for the effects of gesture presence and speech clarity in Analysis 1 and the effect of speech clarity in Analysis 2. The interaction terms as well as the mouth informativeness term were not included in the random structure due to models’ convergence issues. Furthermore, due to singularity fit, models were simplified based on the variance of the random slopes (i.e., the terms that explained the least variance were removed first and then a simplified model was tested). Specifically, we removed the random slopes of gesture presence from the Analysis 1 with incongruent gestures (by participant and by item for the accuracy model, as well as by participant for the RT model) and the random slope of speech clarity by participant from Analysis 2 (accuracy model). By keeping the possibly maximal random structure, we minimized the possibility of Type I errors and ensured a conservative interpretation of the results. To allow convergence, *bobyqa* optimizer was used to maximize the number of iterations each model performed. We also entered word age of acquisition (AoA; Kuperman et al., 2012), log frequency (Brysbaert & New, 2009), number of syllables, and semantic category (i.e., whether the item referred to an action or an object) as control variables.^2^ All continuous predictors were centered on the mean, and all categorical variables were sum-coded (i.e., we compared the deviations from the grand mean [intercept] for a given predictor). We used log transformation of the RT to minimize skewness of the data and then checked for linear regression assumptions: visual inspection of the RT data suggested that the residuals were normally distributed, and the assumption of homoscedasticity was met. There was no multicollinearity (Variance Inflation Factors [VIF] below 1.7). Significance values for the models were obtained using the *lmerTest* package (Kuznetsova et al., 2017) following Luke (2017), with Sattherwaite’s approximation for the RT models and Laplace approximation for the accuracy models. For each model, we additionally calculated conditional *R*^2^ that represents the variance explained by both fixed and random effects following Nakagawa and Schielzeth (2013), as well as Johnson (2014), and using the *MuMIn* package (Bartoń, 2019). Finally, the graphs were created with *sjPlot*(Lüdecke, 2021) and *ggplot2*(Wickham, 2016) packages. The R code and the datasets analyzed in the study are available in the Open Science Framework repository (https://osf.io/gudj6/).

## Results

Here, we report only significant effects and interactions (for the full set of results, see the Supplementary Materials).

### Analysis 1

#### Congruent gestures

The accuracy model revealed a significant main effect of gesture presence (β = −0.389, *SE* = 0.190, *z* = −2.048, *p* = .040) and of speech clarity (β = 0.644, *SE* = 0.204, *z* = 3.156, *p* = .001): Participants made more errors when there were no gestures and when speech was degraded.

**Fig. 3:**
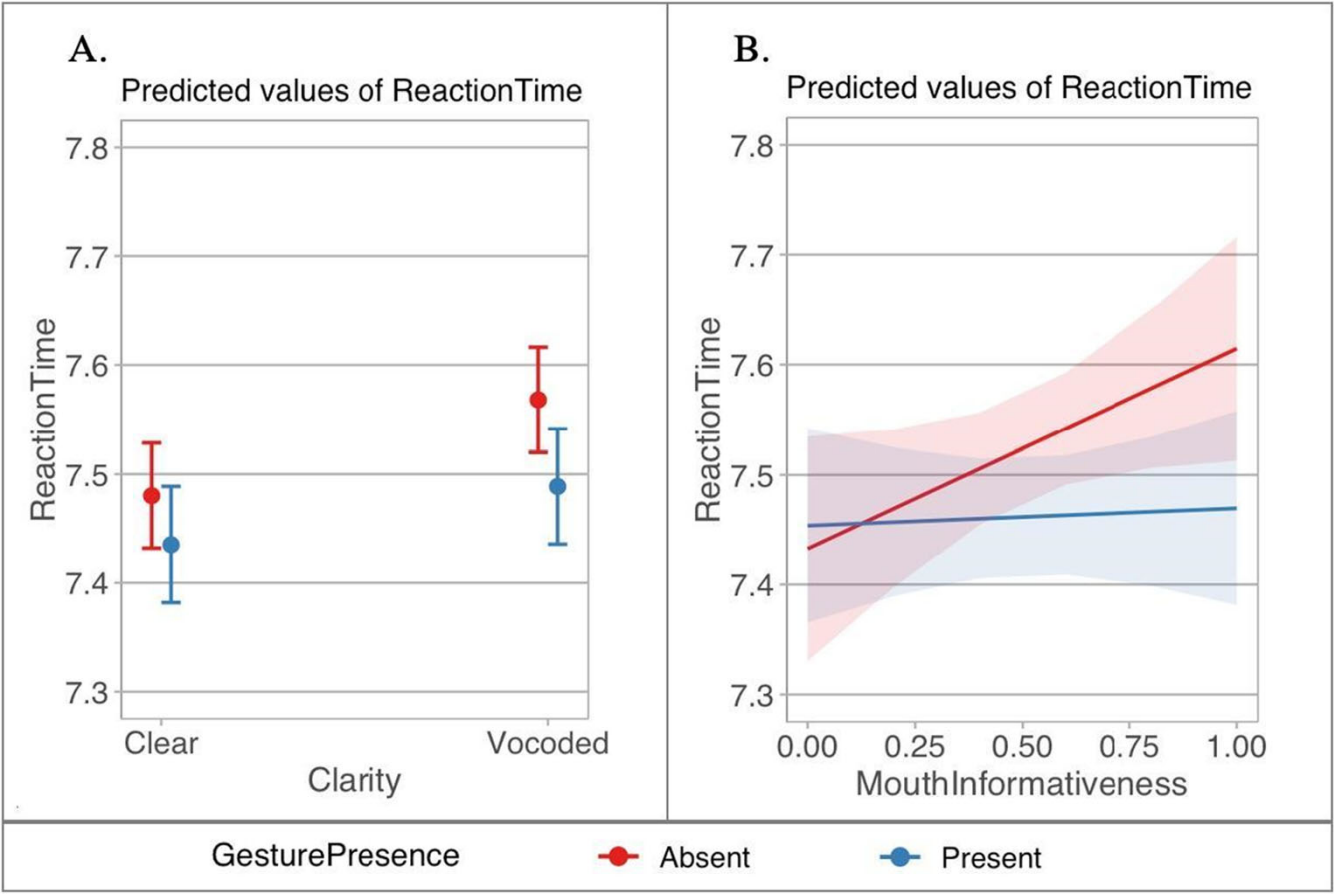
Predicted values of reaction times (log) for Analysis 1 (congruent gestures only). Plot **a** shows an interaction between speech clarity and gesture presence. Plot **b** depicts an interaction between mouth informativeness and gesture presence, with more informative mouth movements being closer to 0. Red color indicates gesture-absent, and blue indicates gesture-present conditions. Error bars represent confidence intervals (95%). (Colour figure online)

In the RT analysis, we found a significant main effect of gesture presence (β = 0.031, *SE* = 0.006, *t* = 4.737, *p* < .001), and of speech clarity (β = −0.035, *SE* = 0.003, *t* = −11.391, *p* < .001): RTs were faster when gestures were present, and when the speech was clear. There was a significant interaction between these variables (β = −0.008, *SE* = 0.002, *t* = −3.645, *p* < .001). Follow-up pairwise comparisons showed that participants were slower in the noise-vocoded condition, especially when gestures were absent (*p*s < .004; see Fig. 3a). The interaction between gesture presence and mouth informativeness was marginal (β = 0.082, *SE* = 0.044, *t* = 1.870, *p* = .064): In the absence of gestures, participants were faster when mouth movements were more informative than when they were less informative (while in the presence of gestures mouth informativeness had no effect); maximal mouth informativeness with no gestures had a similar effect to the presence of gestures (see Fig. 3b).

#### Incongruent gestures

In the accuracy data, we found a significant main effect of gesture presence (β = 0.297, *SE* = 0.069, *z* = 4.322, *p* < .001), and of speech clarity (β = 0.911, *SE* = 0.133, *z* = 6.832, *p* < .001): Participants made more errors with incongruent gestures and when the speech was degraded, respectively.

In the RT analysis, there was a significant main effect of speech clarity (β = −0.041, *SE* = 0.005, *t* = −8.832, *p* < .001) with slower RTs for noise-vocoded speech. The effect of mouth informativeness was marginal (β = 0.153, *SE* = 0.081, *t* = 1.871, *p* = .064): Responses were faster for more informative mouth movements.

#### Analysis 2

**Fig. 4:**
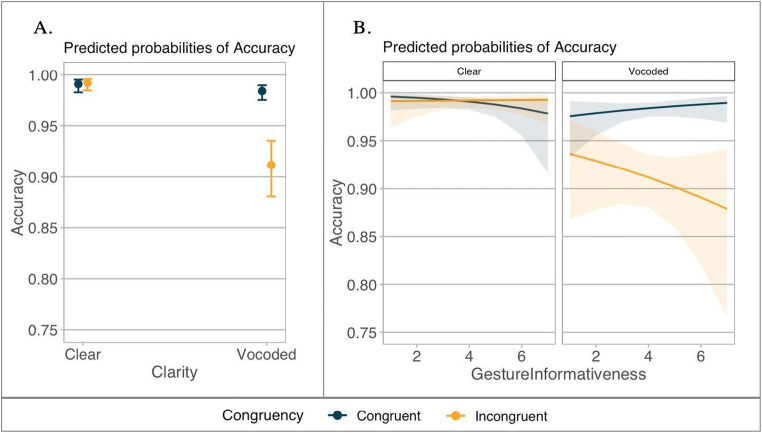
Predicted values of accuracy (proportion) for Analysis 2. The figure shows interactions between congruency and speech clarity (**a**) and congruency, speech clarity, and gesture informativeness (**b**), with more informative gestures being closer to 7. Dark blue represents congruent gestures, and orange represents incongruent gestures. Confidence intervals are set at 95%. (Colour figure online)

In the accuracy analysis, we found a significant main effect of speech clarity (β = 0.765, *SE* = 0.153, *z* = 4.994, *p* < .001), and congruency (β = −0.412, *SE* = 0.097, *z* = −4.234, *p* < .001), with more errors for degraded speech and incongruent pairings, respectively. Their interaction was significant (β = 0.484, *SE* = 0.076, *z* = 6.369, *p* < .001): Participants were especially hindered by vocoding when the gestures were incongruent. There was no difference between clear versus vocoded speech when the gestures were congruent, *p* = .217 (see Fig. 4a). There was also a significant interaction between congruency, speech clarity, and gesture informativeness (β = 0.145, *SE* = 0.072, *z* = 2.025, *p* = .043): Participants performed equally well in both speech clarity conditions when the gesture was congruent, but significantly worse when the noise-vocoded speech was accompanied by highly incongruent gestures (see Fig. 4b).

**Fig. 5:**
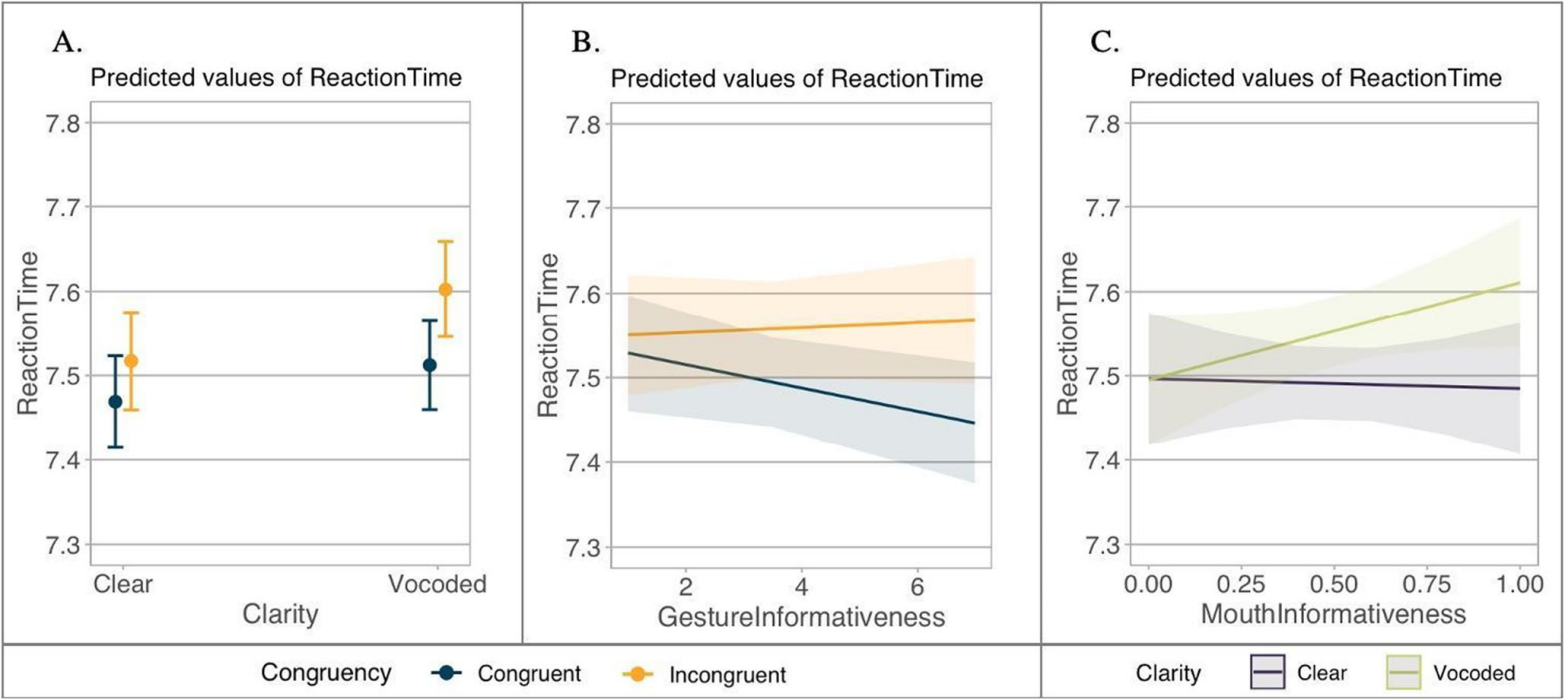
Predicted values of reaction times (log) for Analysis 2. **a** Interaction between speech clarity and congruency. **b** Interaction between congruency and gesture informativeness. **c** Interaction between speech clarity and mouth informativeness. For **a–b**, dark blue represents congruent gestures, whereas orange represents incongruent gestures. For **c**, dark purple refers to clear speech, and green refers to vocoded speech. The larger the value of gesture informativeness, the more informative the gestures are; the smaller the value of mouth informativeness, the more informative the mouth movements are. Confidence intervals are set to 95%

The RT model revealed a significant main effect of speech clarity (β = −0.032, *SE* = 0.004, *t* = −8.232, *p* < .001), with slower RTs for the noise-vocoded speech. We found a significant interaction between congruency and speech clarity (β = −0.010, *SE* = 0.004, *t* = −2.800, *p* = .006). Pairwise comparisons showed that participants were generally slower for vocoded, compared to clear speech for both congruent and incongruent gestures (*p*s < .001). There was no difference between congruent vs. incongruent gesture conditions when the speech was clear (*p* = .441); however, there was a marginal difference in the noise-vocoded condition (*p* = .057) with slower RTs for the incongruent gestures (see Fig. 5a). There was also a significant interaction between congruency and gesture informativeness (β = 0.008, *SE* = 0.003, *t* = 3.147, *p* = .002): Participants responded faster when congruent gestures were more informative (see Fig. 5b). Finally, the interaction between speech clarity and mouth informativeness was also significant (β = −0.066, *SE* = 0.025, *t* = −2.621, *p* = .010): When the speech was degraded, participants were slower for less informative mouth movements, but equally fast when the speech was clear (see Fig. 5c).

## Discussion

We investigated audiovisual word recognition under clear and distorted listening conditions using stimuli for which we have measures of informativeness. Unsurprisingly, subjects were less accurate and slower when the speech was noise vocoded. Replicating previous studies, they were also overall less accurate and slower when gestures were incongruent (e.g., Kelly et al., 2010; McNeill et al., 1994; Vigliocco et al., 2020).

Furthermore, the presence of congruent gestures enhanced word recognition with responses being more accurate and faster particularly when the speech was degraded. Also, faster response times were observed for more informative congruent gestures (relative to incongruent ones) across speech clarity conditions. Conversely, incongruent gestures, especially when they were more informative and accompanied by noise-vocoded speech, led to the least accurate responses.

Informativeness of mouth movements did not have a significant effect across conditions. However, we observed a trend for words with more informative mouth movements having faster RTs when the accompanying gestures were incongruent with the speech. Moreover, mouth informativeness interacted with speech clarity, such that RTs were faster for noise-vocoded words with more informative mouth movements across gesture congruency conditions.

Finally, we found that the two visual cues interact, such that more informative mouth movements speeded up recognition in the absence of gestures and the effect of maximal mouth informativeness was similar to the condition when the gestures were present.

### Iconic gestures and mouth movements in spoken word recognition

In line with previous research, our findings indicate that iconic gestures have a pivotal role in face-to-face communication: They are automatically processed alongside speech and facilitate word recognition, especially for adverse listening conditions (Drijvers & Özyürek, 2017; Holle et al., 2010; Obermeier et al., 2012). Our results also contribute to the growing body of literature on multimodal communication by showing that gestures are particularly useful when highly informative: People derive meaning faster, plausibly because the more information conveyed in gestures, the less ambiguous they are, and thus their conceptual mapping is easier.

Furthermore, when looking at the semantically mismatching trials, people extract information from iconic gestures, even when irrelevant (McNeill et al., 1994). It has been argued that this interference effect reflects automatic and obligatory integration between the two information channels (Kelly et al., 2010). Here, we show that this depends upon gesture informativeness: The clearer the semantic information conveyed in the gestures, the larger the interference. While this result is compatible with an integration account, it may also come about because participants use the information provided by gestures in order to carry out the picture-matching task, rather than speech, as suggested by Vigliocco et al. (2020), to account for the performance of aphasic patients.

Regarding mouth movements, it has been demonstrated that they are part and parcel of both spoken (Sumby & Pollack, 1954) and signed languages (Bank et al., 2016; van de Sande & Crasborn, 2009). Mouth movements are particularly useful in adverse listening conditions (Ma et al., 2009; Reisberg et al., 1987; Ross et al., 2007; Schwartz et al., 2004; Sumby & Pollack, 1954). We extend this result to show that this is crucially the case for more informative mouth movements. More generally, we show that our novel manner of quantifying the amount of information provided by the mouth (mouth informativeness) is useful; it goes beyond manipulating the presence/absence of mouth movements and overcomes the difficulties of existing quantifications based on visemes.

### Dynamic interplay between speech, gesture, and mouth movements

We identified interactions between gesture, mouth informativeness, and clarity, supporting proposals in which auditory and visual cues are dynamically and flexibly weighted during communication (Skipper et al., 2009; Zhang, Frassinelli, et al., 2021b). In one of the first experimental studies looking at audiovisual (including gestures) speech, Drijvers and Özyürek (2017) demonstrated that moderately noise-vocoded speech comprehension was enhanced when the two cues were present, with iconic gestures having a larger effect than mouth movements. Using a completely different task and looking at RTs (as well as accuracy), we replicated and, importantly, extended their results by clarifying how and when gestures and mouth impact word recognition. Specifically, the presence of gestures significantly improved word recognition, irrespectively whether mouth movements were informative or not. This finding can be accounted for in two ways. It could be that participants carried out the task by making a decision as soon as semantic information was accessed either via the speech or via the gesture (whichever came first). In degraded speech conditions, such decisions could be based predominantly on the gesture. This account is in line with our previous findings from aphasic speakers where we found clear evidence for a complementary use of speech and gestures (Vigliocco et al., 2020). Alternatively, the advantage might have come about because together speech and gestures enhanced activation in the semantic system in comparison to speech or gesture alone. Compatible with this latter possibility, we found that when the speech was degraded and accompanied by gestures (either congruent or incongruent) more informative mouth movements helped. This effect may come about because mouth movements facilitate phonological activation of the target word leading to enhanced (when congruent) or reduced (when incongruent) activation at the semantic level. This is in line with Drijvers and Özyürek’s (2017) finding of an enhanced impact on the accuracy of both (rather than single) cues in degraded conditions. The finding, albeit only marginal, of a larger mouth informativeness effect (across clarity conditions) without gestures goes beyond the work of Drijvers and Özyürek (2017), suggesting that the system weights differently the cues using the most useful at any one time.

Zhang, Frassinelli, et al. (2021b) showed that the N400 response evoked by words in context is modulated by the presence of different multimodal cues, such as (both iconic and beat) gestures, prosodic stress, and mouth informativeness. The researchers found that comprehension is enhanced when iconic gestures and more informative mouth movements accompany speech. They explained this finding in terms of eye gaze literature, suggesting that listeners often focus on a speaker’s face during speech–gesture processing (Beattie et al., 2010; Gullberg & Kita, 2009). In parallel, here we found that mouth informativeness had an impact on word recognition across gesture congruency conditions when the speech was degraded, similarly showing that the more information is available, the easier is comprehension.

Overall, our results support the view that both cues contribute to human communication, with iconic gestures playing a more substantial role than mouth movements (Drijvers & Özyürek, 2017). This is because iconic gestures facilitate encoding of the meaning by directly activating semantic features (McNeill, 1992, 2000; Morrel-Samuels & Krauss, 1992). Instead, mouth movements tap into phonological features of words which can then facilitate access to the semantic representations by prediction and constraint (Peelle & Sommers, 2015). Importantly, we also demonstrate that the use of cues depends on their informativeness, suggesting that iconic gestures and mouth movements are dynamically weighted during speech processing.

## Supplementary Information

ESM 1 (PDF 104 kb)

## Data Availability

The data and picture materials are available at https://osf.io/gudj6/

## Footnotes

1 Features are weighted according to their phonological class and their subjective variability. For more information, see: https://github.com/dmort27/panphon

2 Note that word familiarity, phonological neighborhood density, and viewport size (i.e., the size of each participant’s browser window, minus the webpage interface, such as URL bar) were initially considered as control factors but we had to drop them due to missing values and model complexity. An average viewport size was 1,536 × 750.

## Code availability (software application or custom code)

The code is available at https://osf.io/gudj6/
