# Supplementary material for: The role of iconic gestures and mouth movements in face-to-face communication
Source: Psychon Bull Rev. 2021 Oct 20;29(2):600–12. doi: 10.3758/s13423-021-02009-5 (PMC9038814; doi:10.3758/s13423-021-02009-5)
Supplement: Supplementary file 1 — (PDF 104 kb) [file 13423_2021_2009_MOESM1_ESM.pdf]

SUPPLEMENTARY MATERIALS

Outliers

A-priori any analyses, four items were removed to account for the consistency within the dataset: three adjectives (‘cold’, ‘fat’, ‘cold’), and a hyphenated compound noun (‘merry-go-round’), leaving 116 items referring to objects (60) and actions (56) only. Outliers were further identified as: (i) any participant with an accuracy below 3SD or with RT above 3SD from the mean; (ii) any item with an accuracy below chance level (50%) or Reaction Time (RT) above 3SD from the mean; (iii) any trial with RTs greater than 3SD from the mean of all trials; (iv) any trials which had video loading issues signalized by Gorilla.

Analysis 1

For the Congruent dataset, three items and two participants were removed due to RTs longer than 3SD from the mean; one participant was removed due to the accuracy scores below 3SD from the mean; three trials were removed due to loading issues, and 38 trials across participants and items were removed due to the RTs above 3SD from the mean. For the Incongruent dataset, two items and two participants were removed due to the RTs being above 3SD from the mean; one participant performed below 3SD from the accuracy mean; 26 trials were removed due to video loading issues and another 23 trials were removed due to RT longer than 3SD from the mean across all trials. All items were well above chance level (>65%). This left us with 50 participants and 113 items for the Congruent analysis, and 48 participants and 114 items for the Incongruent analysis.

Analysis 2

The outliers were recalculated based on the combined Congruent and Incongruent datasets for the trials in which the gesture was always present. Two items and two participants were above 3SD from the mean based on the RT, and three additional participants scored below 3SD from the mean based on accuracy. Those were subsequently removed from further analysis. Additionally, 37 trials were excluded due to being 3SD above the RT mean based on all the trials across participants. The trials with video-loading issues, as noted above, were also removed, leaving us with a total of 99 participants, 114 items, and 5608 trials to analyze.

Full results

Analysis 1

Table 1

Regression results of Accuracy and Reaction Times (RTs) for the Congruent (A) and Incongruent (B) Gestures.

A.

MIXED-EFFECT REGRESSION RESULTS – CONGRUENT GESTURES

**Dependent variable:**	**Accuracy**					**Reaction Time**					
Model fit:	Maximum likelihood (Laplace Approximation)					Restricted maximum likelihood (Satterthwaite Approximation)					
Conditional R^2	0.134					0.594					
**RANDOM EFFECTS**											
	**Variance**	**SD**				**Variance**	**SD**				
Item (Intercept)	*1.555*	*1.247*				*0.009*	*0.095*				
Gesture Presence	0.710	0.843				0.003	0.058				
Speech Clarity	0.688	0.829				0.000	0.018				
ParticipantID (Intercept)	*0.404*	*0.635*				*0.026*	*0.161*				
Gesture Presence	0.052	0.229				0.000	0.019				
Speech Clarity	0.114	0.338				0.000	0.006				
**FIXED EFFECTS**											
	**Estimate**	**SE**	**z value**	**Pr(>|z|)**		**Estimate**	**SE**	**DF**	**t value**	**Pr(>|z|)**	
(Intercept)	*4.707*	*0.257*	*18.312*	*< 2e-16*	***	*7.493*	*0.024*	*64.220*	*303.528*	*< 2e-16*	***
Gesture Presence	-0.389	0.190	-2.048	0.040	*	0.031	0.006	122.500	4.737	0.000	***
Speech Clarity	0.644	0.204	3.156	0.001	**	-0.035	0.003	56.460	-11.391	0.000	***
Mouth Informativeness	0.841	1.246	0.675	0.499		0.098	0.069	106.800	1.411	0.161	
Gesture Presence:Speech Clarity	-0.114	0.123	-0.930	0.352		-0.008	0.002	4995.00	-3.645	0.000	***
Gesture Presence:Mouth Informativeness	0.771	0.969	0.796	0.426		0.082	0.044	109.300	1.870	0.064	
Speech Clarity:Mouth Informativeness	0.317	0.966	0.329	0.742		0.010	0.022	103.900	0.481	0.631	
Gesture Presence:Speech Clarity: Mouth Informativeness	0.530	0.732	0.724	0.469		0.030	0.017	4895.00	1.724	0.084	
**CONTROL VARIABLES**											
	**Estimate**	**SE**	**z value**	**Pr(>|z|)**		**Estimate**	**SE**	**DF**	**t value**	**Pr(>|z|)**	
Age of Acquisition	-0.200	0.126	-1.588	0.112		0.013	0.007	107.600	1.875	0.063	
Frequency	0.011	0.240	0.047	0.962		-0.008	0.014	106.300	-0.609	0.543	
Number of Syllables	0.233	0.329	0.708	0.479		0.037	0.018	106.500	2.100	0.038	*
Semantic Category	-0.450	0.174	-2.581	0.009	**	-0.011	0.010	106.600	-1.090	0.278	

Note: Reference levels: 'Clear' for Speech Clarity, 'Absent' for Gesture Presence, and 'Action' for Semantic Category. *0.05; **p<0.01; ***p<0.001

B.

MIXED-EFFECT REGRESSION RESULTS – INCONGRUENT GESTURES

**Dependent variable:**	**Accuracy**					**Reaction Time**					
Model fit:	Maximum likelihood (Lapplace Approximation)					Restricted maximum likelihood (Satterthwaite Approximation)					
Conditional R^2	0.154					0.500					
**RANDOM EFFECTS**											
	**Variance**	**SD**				**Variance**	**SD**				
Item (Intercept)	*0.813*	*0.901*				*0.012*	*0.109*				
Gesture Presence	n/a due to singularity					0.005	0.071				
Speech Clarity	0.063	0.251				0.000	0.012				
ParticipantID (Intercept)	*0.753*	*0.868*				*0.030*	*0.173*				
Gesture Presence	n/a due to singularity					n/a due to singularity					
Speech Clarity	0.143	0.379				0.000	0.021				
**FIXED EFFECTS**											
	**Estimate**	**SE**	**z value**	**Pr(>|z|)**		**Estimate**	**SE**	**DF**	**t value**	**Pr(>|z|)**	
(Intercept)	*3.454*	*0.194*	*17.824*	*< 2e-16*	***	*7.567*	*0.027*	*61.860*	*277.558*	*< 2e-16*	***
Gesture Presence	0.297	0.069	4.322	0.000	***	-0.012	0.007	110.000	-1.685	0.095	
Speech Clarity	0.911	0.133	6.832	0.000	***	-0.041	0.005	42.930	-8.832	0.000	***
Mouth Informativeness	-0.698	0.871	-0.802	0.432		0.153	0.081	107.800	1.871	0.064	
Gesture Presence:Speech Clarity	-0.046	0.068	-0.668	0.504		0.003	0.003	4677.00	0.808	0.419	
Gesture Presence:Mouth Informativeness	0.542	0.548	0.989	0.323		0.094	0.055	110.600	1.692	0.094	
Speech Clarity:Mouth Informativeness	0.345	0.584	-0.517	0.554		-0.034	0.026	103.900	-1.315	0.191	
Gesture Presence:Speech Clarity: Mouth Informativeness	-0.282	0.545	-0.517	0.605		0.044	0.025	4722.00	1.804	0.071	
**CONTROL VARIABLES**											
	**Estimate**	**SE**	**z value**	**Pr(>|z|)**		**Estimate**	**SE**	**DF**	**t value**	**Pr(>|z|)**	
Age of Acquisition	-0.229	0.093	-2.456	0.014	*	0.004	0.009	108.000	0.485	0.629	
Frequency	-0.298	0.182	-1.634	0.102		-0.018	0.018	107.500	-0.973	0.333	
Number of Syllables	0.227	0.220	1.031	0.303		0.044	0.022	107.200	2.050	0.043	*
Semantic Category	-0.297	0.124	-2.390	0.017	*	-0.009	0.012	107.200	-0.734	0.465	

Note: Reference levels: 'Clear' for Speech Clarity, 'Absent' for Gesture Presence, and 'Action' for Semantic Category. *0.05; **p<0.01; ***p<0.001

Analysis 2

Table 2

Regression results of Accuracy and Reaction Times (RTs) for the combined Congruent and Incongruent Gestures.

MIXED-EFFECT REGRESSION RESULTS – CONGRUENT & INCONGRUENT GESTURES

**Dependent variable:**	**Accuracy**	**Reaction Time**
Model fit:	Maximum likelihood (Laplace Approximation)	Restricted maximum likelihood (Satterthwaite Approximation)
Conditional R^2	0.137	0.466

RANDOM EFFECTS

	**Variance**	**SD**	**Variance**	**SD**
Item (Intercept)	*1.299*	*1.401*	*0.007*	*0.081*
Speech Clarity	0.425	0.652	0.000	0.011
ParticipantID (Intercept)	*0.322*	*0.567*	*0.034*	*0.184*
Speech Clarity	n/a due to singularity		0.000	0.020

FIXED EFFECTS

	**Estimate**	**SE**	**z value**	**Pr(>|z|)**		**Estimate**	**SE**	**DF**	**z value**	**Pr(>|z|)**	
(Intercept)	*3.985*	*0.193*	*20.682*	*< 2e-16*	***	*7.525*	*0.020*	*126.50*	*370.685*	*< 2e-16*	***
Congruency	-0.412	0.097	-4.234	0.000	***	0.035	0.019	96.890	1.834	0.070	
Speech Clarity	0.765	0.153	4.994	0.000	***	-0.032	0.004	70.010	-8.232	0.000	***
Mouth Informativeness	-0.514	1.110	-0.463	0.643		0.053	0.062	103.90	0.859	0.392	
Gesture Informativeness	-0.059	0.131	-0.447	0.655		-0.005	0.007	107.00	-0.727	0.469	
Congrueny:Speech Clarity	0.484	0.076	6.369	0.000	***	-0.010	0.004	94.000	-2.800	0.006	**
Congrueny:Mouth Informativeness	-1.068	0.632	-1.690	0.091		0.013	0.023	4898.0	0.557	0.577	
Speech Clarity:Mouth Informativeness	0.675	0.859	0.786	0.432		-0.066	0.025	109.10	-2.621	0.010	*
Congrueny:Gesture Informativeness	0.015	0.072	0.205	0.838		0.008	0.003	4971.0	3.147	0.002	**
Speech Clarity:Gesture Informativeness	-0.071	0.099	-0.724	0.469		-0.001	0.003	108.10	-0.284	0.777	
Mouth Informativeness:Gesture Informativeness	-1.099	0.919	-1.196	0.232		-0.068	0.050	104.00	-1.360	0.177	
Congrueny:Speech Clarity:Mouth Informativeness	0.157	0.632	0.249	0.804		-0.027	0.024	4934.0	-1.121	0.262	
Congrueny:Speech Clarity:Gesture Informativeness	0.145	0.072	2.025	0.043	*	-0.001	0.003	4830.0	-0.447	0.655	
Congrueny:Mouth Informativeness:Gesture Informativeness	0.386	0.483	0.799	0.424		0.033	0.019	5007.0	1.746	0.081	
Speech Clarity:Mouth Informativeness:Gesture Informativeness	0.241	0.716	0.336	0.737		-0.029	0.020	105.30	-1.439	0.153	

CONTROL VARIABLES

	**Estimate**	**SE**	**z value**	**Pr(>|z|)**		**Estimate**	**SE**	**DF**	**z value**	**Pr(>|z|)**	
Age of Acquisition	-0.278	0.104	-2.664	0.008	**	0.013	0.007	105.10	1.809	0.073	
Frequency	-0.214	0.207	-1.035	0.301		0.005	0.014	104.00	0.345	0.731	
Number of Syllables	0.157	0.250	0.628	0.530		0.038	0.017	103.70	2.269	0.025	*
Semantic Category	-0.333	0.142	-2.345	0.019	*	-0.004	0.010	103.70	-0.440	0.661	

Note: Reference levels: 'Clear' for Speech Clarity, 'Incongruent' for Congruency, and 'Action' for Semantic Category. *0.05; **p<0.01; ***p<0.001
